# phbuilder: A Tool for Efficiently Setting up Constant pH Molecular Dynamics Simulations in GROMACS

**DOI:** 10.1021/acs.jcim.3c01313

**Published:** 2024-01-12

**Authors:** Anton Jansen, Noora Aho, Gerrit Groenhof, Pavel Buslaev, Berk Hess

**Affiliations:** † Department of Applied Physics and Swedish e-Science Research Center, Science for Life Laboratory, KTH Royal Institute of Technology, 100 44 Stockholm, Sweden; ‡ Nanoscience Center and Department of Chemistry, University of Jyväskylä, 40014 Jyväskylä, Finland

## Abstract

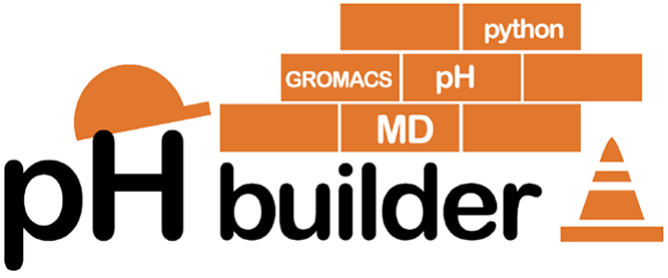

Constant pH molecular dynamics (MD) is a powerful technique that allows the protonation state of residues to change dynamically, thereby enabling the study of pH dependence in a manner that has not been possible before. Recently, a constant pH implementation was incorporated into the GROMACS MD package. Although this implementation provides good accuracy and performance, manual modification and the preparation of simulation input files are required, which can be complicated, tedious, and prone to errors. To simplify and automate the setup process, we present phbuilder, a tool that automatically prepares constant pH MD simulations for GROMACS by modifying the input structure and topology as well as generating the necessary parameter files. phbuilder can prepare constant pH simulations from both initial structures and existing simulation systems, and it also provides functionality for performing titrations and single-site parametrizations of new titratable group types. The tool is freely available at www.gitlab.com/gromacs-constantph. We anticipate that phbuilder will make constant pH simulations easier to set up, thereby making them more accessible to the GROMACS user community.

## Introduction

Molecular dynamics (MD) simulations of biomolecular systems are nowadays widely applied to study a broad range of research questions, from protein folding^1^ to drug design.^2^ While parameters such as temperature and pressure are routinely taken into account in MD simulations, pH, a critical parameter influencing the structure, dynamics, and function of many biomolecules,^3^ cannot be controlled in standard MD simulations. To overcome this limitation and capture the effects of pH, constant pH simulation methods have been developed.^4−28^ We have recently presented an efficient implementation of constant pH for the GROMACS MD package^29^ based on the λ-dynamics approach first introduced by Brooks and co-workers in 1996.^30^ Similar implementations are also available in other popular MD software packages such as CHARMM^22^ and Amber.^26^

While progress has been made in refining the methodological aspects of constant pH MD, the setup of such simulations remains complicated. Setup requires several steps, including preparation of the initial structure and modifying the topology and parameter files. Structure preparation requires adding dummy hydrogens to all titratable groups in the system; topology modification requires assigning charges for all protonation states of titratable groups, and modification of the parameter files includes the addition of parameters specific for constant pH MD simulations. Although these are general steps for constant pH MD,^31^ the implementation details of such setup steps can differ significantly between MD engines and often require time-consuming and error-prone manual modifications. Setting up constant pH MD also requires a deep understanding of the system’s chemistry and force field formatting, which are also specific to a particular MD engine.

Constant pH MD simulations are not the only type of simulation that is difficult to set up. Over the years, several tools have been developed to aid in the setup of MD simulations: CHARMM-GUI^32^ is a web environment that allows the user to generate input files of a wide range of biomolecular systems for various simulation approaches. PACKMOL^33^ can generate structures of complex biomolecular systems, while BioSimSpace^34^ is a Python framework that allows one to set up and run simulations with different software engines. Several tools were also developed to automate preparation steps for free energy simulations.^35−40^ Such tools simplify the setup process by automating the time-consuming and error-prone generation of simulation input files. However, there is currently no such tool for automatically setting up constant pH simulations for GROMACS.

To bridge this gap, we have developed phbuilder, a Python-based command line tool that automates the constant pH specific preparation steps when setting up MD simulations. phbuilder generates the required topology and parameter files, and it also adds the appropriate number of ions and neutralizing buffer particles^27,28^ to keep the simulation box neutral during the constant pH MD run. To demonstrate the tool, we used it to prepare constant pH MD simulations starting from both separate structure files and existing simulation systems. Additionally, phbuilder provides functionality through the inclusion of stand-alone Python scripts for setting up titrations and parametrizations for new titratable group types. Currently, only parametrization of single-site titratable groups is supported. Multisite parametrization will be addressed in a future work and merged into phbuilder when ready. We demonstrate the parametrization feature by parametrizing a single-site model of arginine.

## Methods

### phbuilder Implementation

**Figure 1:**
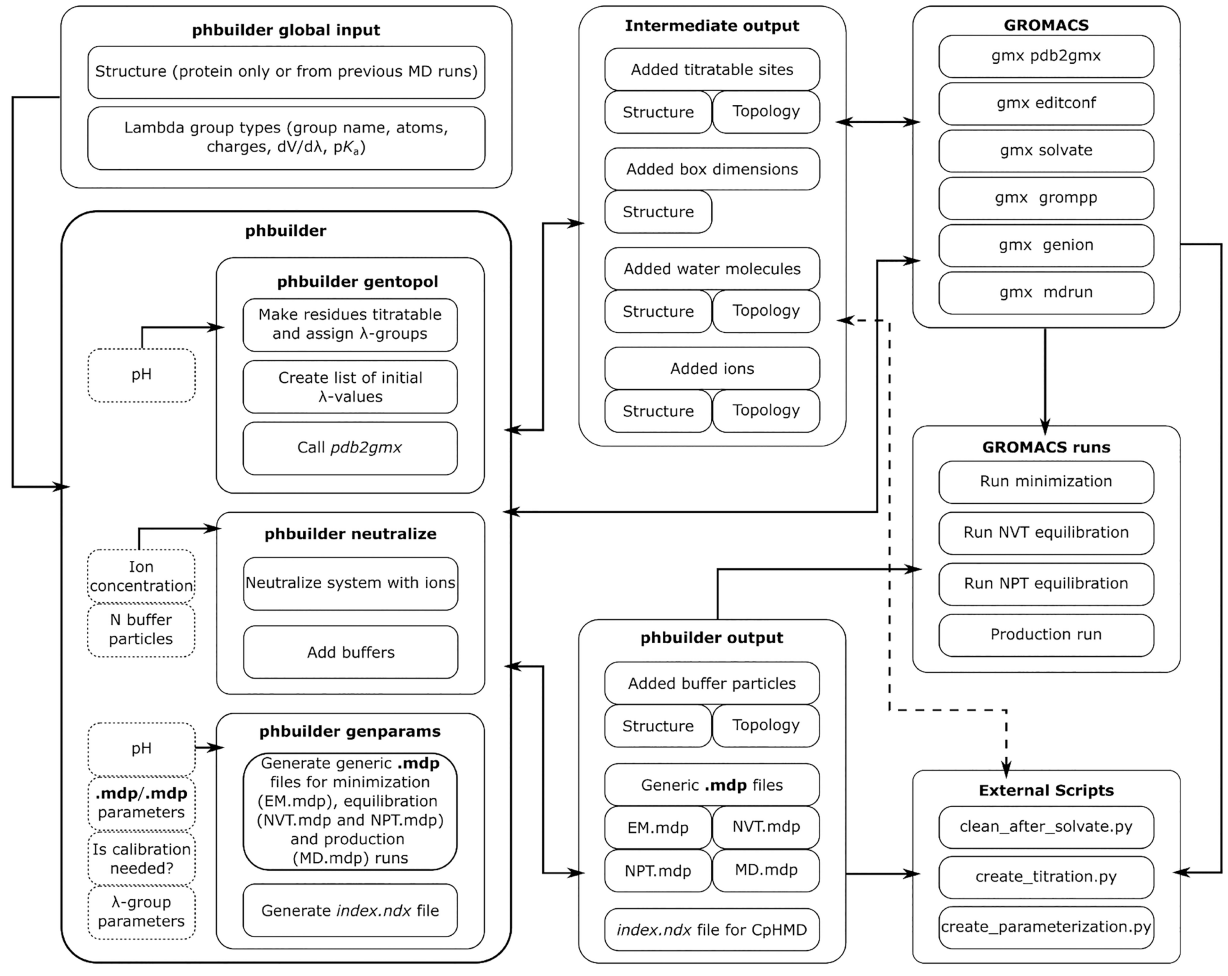
Schematic of the internal structure of phbuilder, as well as the movement of various input and output files and the external GROMACS programs and scripts utilized. phbuilder consists of three tools: **gentopol**, **neutralize**, and **genparams**. **gentopol** prepares the structure and topology files. **neutralize** ensures the appropriate number of ions and buffer particles for a net-neutral system. **genparams** generates the .mdp and .ndx parameter files required for running constant pH MD in GROMACS.

phbuilder is a Python-based command line tool that assists in setting up and performing constant pH MD simulations with GROMACS.^27−29^ Both the tool and a user manual (including tutorials) are available at www.gitlab.com/gromacs-constantph. In simple cases such as a globular protein in a box of water, the only required input is a structure file (.pdb or .gro) and the desired simulation pH. The input can come from either an initial structure file or a prepared simulation system. During the setup process, the user can optionally provide additional parameters, such as the desired ion concentration and the number of buffer particles to add. When the structure file is processed, all residue types should be available in the force field. If all residue types are not available, then the user will be prompted to manually provide the missing topologies. Currently, the GROMACS constant pH implementation only supports a modified version of the CHARMM36m force field,^41^ which includes dihedral modifications for improved sampling of the dihedral angles of the titratable groups.^28^ In addition to system-specific input, phbuilder reads from a global parameter file, lambdagrouptypes.dat, which stores the topology information on the titratable group types. The details of the lambdagrouptypes.dat organization and modification are discussed in the following sections. With the aforementioned input, phbuilder then generates the topology, index, and generic .mdp files required for running constant pH simulations. Scripts for the automation of titration and parametrization runs are also provided.

phbuilder consists of three tools: **gentopol**, **neutralize**, and **genparams**, as presented in Figure 1. First, **gentopol** makes the residues selected by the user titratable and (re)generates the topology accordingly using gmx pdb2gmx. **gentopol** also generates a list of initial λ-values for each titratable group (based on the pH value set by the user) and saves the correspondence between λ-coordinates and actual residues in the system. If necessary, the structure resulting from **gentopol** can be solvated using gmx editconf and gmx solvate tools.

Because gmx solvate can accidentally place water molecules inside the protein’s hydrophobic core, we provide a Python script, clean_after_solvate.py, which can remove those misplaced water molecules with one of two algorithms implemented:

1. Number of water molecules around other waters. This algorithm is the default. For each water molecule within the specified cutoff *R*_c_ of non-solvent molecules (default value for *R*_c_ is 5 Å), the number of water atoms *N*_atoms^HOH^_ within a sphere of radius *R* (default value is 10 Å) is calculated. For water molecules outside of the protein, this number is large (around 400), while for water molecules inside the protein, this number is smaller than *N*_cutoff_ = 120 (Figure S1). The latter water molecules are thus removed. The default values for *R*_c_, *R*, and *N*_cutoff_ might not be optimal for every system. In case of a severely curved protein shape, it might be useful to increase *R*_c_ to get more statistics on water molecules that are far from the protein. In case of smaller simulation system sizes, reducing *R* is meaningful. But in general, the default values for *R* and *R*_c_ are reasonable. As for *N*_cutoff_, the default value is based on the distribution of the number of water molecules around selected waters. As demonstrated in Figure S1, *N*_cutoff_ = 120 clearly separates the majority of water molecules, which are surrounded by many water molecules, from a few waters, which have only a few other water molecules around. We recommend that users test optimal values for the system of interest themselves.
2. Number of water molecules among nearest neighbors. For each water molecule within the specified cutoff *R*_c_ of non-solvent molecules, the *N*_neighbors_ nearest residues are detected first (default value of *N*_neighbors_ is 20). Then, the number of water molecules around those neighbors is calculated. For water molecules far from the protein, almost all the neighbors are other water molecules, while buried residues will have only a few neighbor water molecules (Figure S1C,D). By default, water is removed if the number of water molecules in its neighbor list is less than 3. Similar to the first method, the default *R*_c_ and *N*_neighbors_ values should be reasonable for most systems. The default cutoff value was selected, as it clearly separates water molecules that are surrounded by other water molecules from those surrounded mainly by the protein (Figure S1C). We recommend that users test optimal parameters for the system of interest themselves.

The user decides which of these two methods is used for removing misplaced water molecules.

The **neutralize** tool ensures a net-neutral charge in the simulation system. First, it adds ions to make the system neutral for the configured initial state. It can also add ions at a given concentration if specified by the user. Second, **neutralize** adds buffer particles, which will compensate for charge fluctuations during constant pH MD simulations.^27,28,42^ By default, the **neutralize** tool enforces a minimum placement distance of 0.6 nm from non-solvent molecules when replacing the solvent with ions and buffer particles. If it is observed that the ions or buffer particles are (re)placed in pockets inside a protein, this may be remedied by manually increasing the minimum placement distance through the –rmin flag. By default, **neutralize** will add as many buffer particles as the total number of titratable sites *N*_sites_ in the system. However, the number of buffers may also be manually set by the user. The general recommendation is that the λ-value of the buffer should be close to 0.5 (which corresponds to zero charge for the buffers) during the entire simulation.^28^ While this can be achieved by increasing the total number of buffers to up to twice the number of titratable sites, we nevertheless recommended using smaller buffer concentrations in order to not significantly alter the solvent properties. Thus, the optimal buffer concentration can be selected in the range from , the average charge fluctuation,^43^ to 2*N*_sites_, the maximal possible charge fluctuation, divided by the maximal buffer charge (0.5).

The **genparams** tool generates the .mdp files required for running constant pH simulations with GROMACS. Included in the .mdp files are general constant pH parameters, a description of the titratable group types used in the simulation, and a mapping between atoms and titratable group types (see also the original GROMACS constant pH implementation publication^27^ and the online manual).^44^ General constant pH parameters can be provided by the user together with standard .mdp parameters. Entries for titratable group type descriptions are based on the lambdagrouptypes.dat file, and the mapping between atoms and titratable group types is generated based on the topology and lambdagrouptypes.dat. In addition to the .mdp entries, the indices of atoms that are part of titratable groups are added to an index.ndx file. **gentopol** also sets up the initial λ-values for all λ-coordinates in the .mdp file based on the pH set by the user, and it provides the user the possibility to set up the barrier of the biasing potential^27^ for each λ-coordinate. The lambdagrouptypes.dat file defines the parameters of titratable groups, the path to the GROMACS constant pH installation, and the force field and water model to use. The entries for a single-site titratable group are organized as follows:

[ ASPT ]

incl = ASP ASP1 ASPH ASPP ASH

atoms = CB CG OD1 OD2 HD2

qqA = −0.21 0.75 −0.55 −0.61 0.44

pKa_1 = 3.65

qqB_1 = −0.28 0.62 −0.76 −0.76 0.00

dvdl_1 = −54.078 −144.280 278.550 −146.030 −554.270 44.621

First, the group name is provided inside the square brackets, followed by alternative names, which will be used to search for residues that phbuilder should make titratable. Next, the names of atoms that have different charges in different protonation states of the residue are given. The atomic charges of two protonation states should be provided as qqA and qqB_1 entries. The order of the charges should correspond to the order of the atom names. Then, the p*K*_a_ value of the titratable group in water as well as the coefficients of the polynomial fit of the correction potential *V*^MM^ should be provided. For more details on *V*^MM^, please check the GROMACS constant pH publication and repository.^27^

For titratable groups described with the multisite representation (such as histidine with three protonation states), the details of each protonation state should be listed:

[ HSPT ]

incl = HIS HIS1 HISA HISB HISH HISD HISE HISP HSD HSE HSP

atoms = CB CD2 HD2 CG NE2 HE2 ND1 HD1 CE1 HE1

qqA = 0.00 0.00 0.00 0.00 0.00 0.00 0.00 0.00 0.00 0.00

pKa_1 = 0.0

qqB_1 = −0.05 0.19 0.13 0.19 −0.51 0.44 −0.51 0.44 0.32 0.18

dvdl_1 = −3015.4 −13444.1 −34779. ...

pKa_2 = 6.53

qqB_2 = −0.08 −0.05 0.09 0.22 −0.36 0.32 −0.7 0.0 0.25 0.13

dvdl_2 = −1695.49 −8107.68 −22614.3 ...

pKa_3 = 6.92

qqB_3 = −0.09 0.22 0.1 −0.05 −0.7 0.0 −0.36 0.32 0.25 0.13

dvdl_3 = −111.044 −768.566 −9092.12 ...

The multisite representation allows for the simulation of chemically coupled titratable sites.^27,28^ In general, if a group can be found in *N* different protonation states, the multisite representation will include *N* + 1 states: one artificial common state and *N* physical states of the group. The charges for the common state (which are usually zeros) should be provided as qqA. For each physical state, *i*, the corresponding p*K*_a_ values, charges, and polynomial coefficients of the correction potential should be provided as pKa_i, qqB_i, and dvdl_i, respectively. The full details on how to select the correct p*K*_a_ values and polynomial coefficients can be found in our previous GROMACS constant pH publications.^27,28^

After the structure and parameters for the constant pH MD simulation are automatically generated by phbuilder (and modified by the user if needed), the system needs to be minimized and equilibrated, after which the constant pH MD simulation can be started. In addition to the tools described previously, three external scripts are provided: (1) create_parameterization.py, to set up a parametrization of novel single-site compounds; (2) fit_parameterization.py; to compute the polynomial coefficients of the *V*^MM^ fit from the parametrization runs; and (3) create_titration.py, to set up titration runs of the equilibrated systems. Both the titration and parametrization require multiple constant pH MD simulations of the same system with different input conditions (pH in case of titration, fixed λ-values in case of parametrization). The scripts create_parameterization.py and create_titration.py prepare directories and input .mdp files for those runs automatically. fit_parameterization.py calculates the optimal *V*^MM^ fit and provides new entries for lambdagrouptypes.dat and .mdp files for this group. Details of the procedure for obtaining *V*^MM^ are provided in the next section.

### Parameterization with phbuilder

The results of constant pH MD simulations depend on the accuracy of the correction potential *V*^MM^, which compensates for the quantum mechanical contributions to the proton affinity that are missing from the force field description. These correction potentials are specific for each titratable group and are obtained as polynomial fits to the deprotonation free energy profiles, as we discussed in detail in our previous work.^27,28^ Originally, the derivation of the correction potential *V*^MM^ was based on thermodynamic integration (TI).^45^ However, TI can be inefficient and can lead to an inaccurate fit of *V*^MM^.^46,47^ To avoid these issues, we propose a new two-step scheme for the parametrization of titratable groups: first, we run fast, but not very accurate, TI to obtain an approximated fit of *V*^MM^; then, we reweigh *V*^MM^ based on long sampling simulations of the group simulated at p*K*_a_ = pH with the biasing barrier set to 0. Under such conditions, the distribution of the λ-coordinate should be flat if *V*^MM^ is fitted accurately.^27,28^ However, after a fast TI step, this might not be the case. If the potential derived from the fast TI step is insufficient, the correction to the *V*^MM^ can be calculated using Boltzmann inversion:
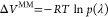
1where *p*(λ) is the probability distribution of λ-coordinates obtained from the sampling runs. After the reweighting step and performing the sampling simulations of arginine at p*K*_a_ = pH with the biasing barrier set to 0, the obtained distributions of the λ-coordinates are flat (Figure S2). phbuilder provides the functionality to perform both steps and outputs the appropriate entries for the lambdagrouptypes.dat and .mdp files, thereby making the parametrization of new molecules more straightforward. However, we would like to emphasize that parametrization is currently only supported for single-site titratable groups. We are currently developing a version for the parametrization of multisite titratable groups, which we will separately report and include in phbuilder when it is ready.

### Simulation Details

**Figure 2:**
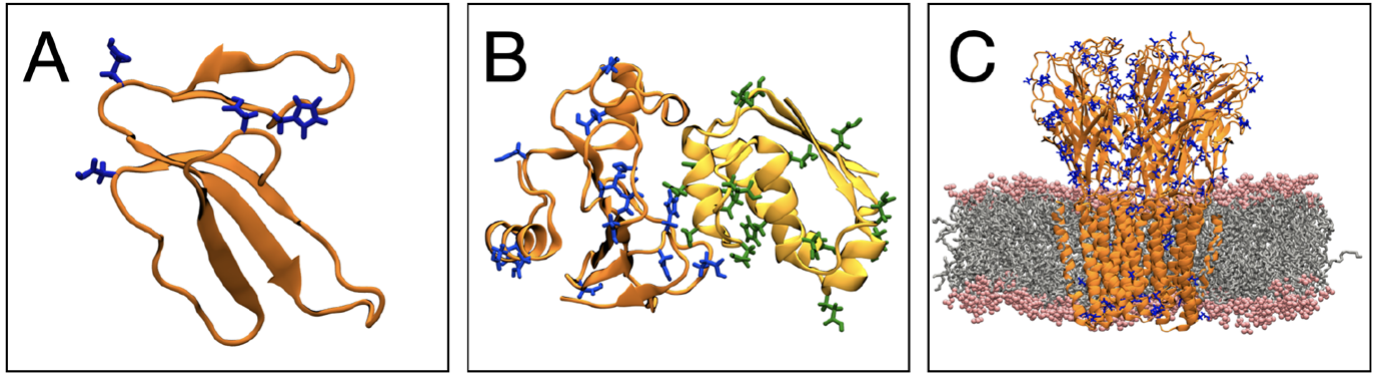
phbuilder was used to set up constant pH MD simulations for a number of different systems: (A) cardiotoxin V (PDB ID: 1CVO),^48^ (B) the Barnase–Barstar protein complex (PDB ID: 1BRS),^49^ and (C) the GLIC ion channel (PDB ID: 6ZGD).^50^ The titratable residues (Asp, Glu, and His residues) are shown as blue sticks in cardiotoxin, GLIC, and Barnase–Barstar chain A and as green sticks in Barnase–Barstar chain B.

To demonstrate the tool’s versatility, we set up constant pH MD simulations for the following systems: (1) cardiotoxin V (PDB ID: 1CVO),^48^ (2) the Barnase–Barstar complex (PDB ID: 1BRS),^49^ and (3) the proton-gated ion channel GLIC (PDB ID: 6ZGD).^50^ The structures of these proteins are shown in Figure 2. For cardiotoxin V, we used phbuilder to set up the system starting from a .pdb file from the Protein Data Bank^51^ as well as from the prepared simulation system. For the Barnase–Barstar complex, phbuilder was used to demonstrate the preparation of a system with multiple chains from a .pdb file. We also used the tool to set up a constant pH MD simulation for an existing GLIC membrane system.

Additionally, phbuilder was utilized to parametrize the coefficients of the *V*^MM^ polynomial fit for arginine. Arginine has three possible deprotonated states^52^ and ideally requires a multisite description. However, here we only considered a single site of *N*_η_1__, as we want to demonstrate setting up parametrizations for single-site groups, which are the most common. For all cases (starting from a .pdb file, starting from a prepared system, and parametrization), we used the same force field and simulation parameters, which are described next.

For all simulation systems, the interactions were modeled using the CHARMM36 all-atom force field^41^ with dihedral modifications for improved sampling of the amino acid side-chain rotamers.^28^ This modified force field already contains the entries for the titratable glutamic acid (Glu), aspartic acid (Asp), and histidine (His) residues as well as the buffer particles.^27,28^

To prepare the system for cardiotoxin V and the Barnase–Barstar complex (either prior to or while applying phbuilder), the following procedure was used: (1) the corresponding .pdb file was obtained from the Protein Data Bank.^51^ (2) The protein was placed in a cubic box with a 2.0 nm distance between the protein and the box. (3) The system was solvated with ∼17000 CHARMM TIP3P^53,54^ waters for cardiotoxin V and ∼31000 for Barnase–Barstar. Finally, (4) a 0.1 M concentration of Na^+^ and Cl^–^ ions were added to neutralize the system. The system for GLIC (prior to applying phbuilder) was prepared using CHARMM-GUI,^32^ and it contained ∼62000 CHARMM TIP3P water molecules,^53,54^ 489 POPC (1-palmitoyl-2-oleoylphosphatidylcholine) lipids, and a 0.15 M concentration of Na^+^ and Cl^–^ ions. Arginine was simulated as an Ala–Arg–Ala tripeptide with capped termini. The box size was ∼5 × 5 × 5 nm^3^, and the system contained ∼5000 CHARMM TIP3P water molecules^53,54^ and a 0.15 M concentration of Na^+^ and Cl^–^ ions. For all of the simulated systems, the input configurations, together with the topology and run parameters, are provided (see the Data and Software Availability section).

In all of the simulations, the same simulation parameters that were recommended for the CHARMM force field were used. The Coulomb interactions were computed with the particle mesh Ewald (PME) method^55,56^ with a real-space cutoff of 1.2 nm and a grid spacing of 0.14 nm. The van der Waals interactions were modeled with the Lennard-Jones potential and smoothly switched to zero in the range from 1.0 to 1.2 nm. A constant temperature was maintained at 300 K using the v-rescale thermostat^57^ with a time constant of 0.5 ps^–1^, and a constant pressure was maintained at 1 bar using the c-rescale barostat^58^ with a relaxation time of 5.0 ps. For GLIC, a semi-isotropic barostat was used. In all simulations, the leapfrog integrator and a time step of 2 fs was used. The bond lengths to hydrogen atoms were constrained with the LINCS algorithm,^59^ and the internal degrees of freedom of the water molecules were constrained with the SETTLE algorithm.^60^

Next, details of the constant pH MD simulations constructed and performed by applying phbuilder are given. For the protein systems, the aspartic acid (Asp), glutamic acid (Glu), and histidine (His) residues were considered as titratable groups. The reference p*K*_a_ values were 3.65 and 4.25 for Asp and Glu, respectively, and 6.53 and 6.92 for His HSD (singly protonated at the N_δ_) and His HSE (singly protonated at the N_ϵ_), respectively. For the λ-particles, the mass was set to 5 atomic units, and the temperature was maintained at 300 K by using a separate v-rescale thermostat with a time constant of 2.0 ps^–1^. The barrier height of the biasing potential barrier was set at 7.5 kJ/mol.

After the construction of the systems, the potential energy of each system was minimized by using the steepest descent algorithm, followed by 10 ps of NVT equilibration and 10 ps of NPT equilibration. During these simulations, the λ-coordinates of all titratable groups were kept constant. Then, a 10 ns constant pH simulation was performed for cardiotoxin V from a .pdb file at pH = 4 and from an existing system at pH = 4, for Barnase–Barstar at pH = 7, and for GLIC at pH = 4. For the parametrization of arginine, 1 ns simulations with λ-coordinates fixed at values ranging from −0.1 to 1.1 with 0.1 steps were first run to obtain an initial guess for the *V*^MM^. Then, 10 replicas of 100 ns simulations with pH = p*K*_a_ and the biasing barrier set to 0 were run. From those sampling simulations, new coefficients for the *V*^MM^ polynomial fit were calculated, and another 10 replicas of 100 ns simulations with pH = p*K*_a_ and the biasing barrier set to 0 with updated *V*^MM^ were run.

## Results and Discussion

Here, we demonstrate the tool’s efficacy by preparing constant pH MD simulations for initial structure files, for structures from existing MD simulation systems, and for the parametrization of a new titratable group. All systems were subsequently simulated for 10 ns to demonstrate that the system setup was correct.

### Setting up Simulations from Protein Structure

**Figure 3:**
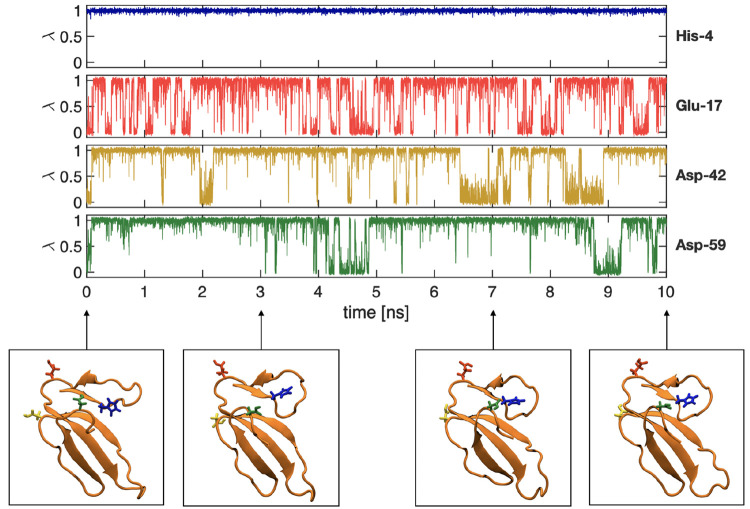
Evolution of the λ-coordinates over time, along with snapshots of the trajectory for a 10 ns constant pH MD simulation of cardiotoxin V (PDB ID: 1CVO)^48^ at pH = 4. The upper panel presents the λ-coordinates as a function of the simulation time for His-4, Glu-17, Asp-42, and Asp-59, and the lower panels show snapshots at 0, 3, 7, and 10 ns. In the snapshots, the titratable groups are shown with the stick representation, and the colors of the groups match those of the λ-coordinate plots.

Two constant pH MD simulations were set up from initial protein structures. These were cardiotoxin V (four titratable groups) and the Barnase–Barstar complex (30 titratable groups). phbuilder successfully set up both of these systems. We subsequently performed 10 ns simulation runs, and the protonation states of the titratable groups evolved together with the atomic coordinates (Figure 3 for cardiotoxin V and Figure S3 for the Barnase–Barstar complex).

### Setting up Simulations for a Prepared System

As mentioned previously, phbuilder can also convert existing simulations to constant pH simulations. To demonstrate this feature, we set up constant pH MD simulations based on an existing cardiotoxin V system and from a membrane protein that was prepared using CHARMM-GUI.^32^ We used CHARMM-GUI as an example of a preparation workflow for membrane proteins, but phbuilder can work with any workflow that prepares structures for MD simulations, such as inflategro^61^ or g_membed.^62^

We first set up and performed a 10 ns constant pH simulation of cardiotoxin V from an existing simulation system and made a comparison to the initial cardiotoxin V simulation performed earlier. In both cases, we found that histidine was always protonated, while aspartic acids were predominantly deprotonated and underwent only a few transitions. Glutamic acid showed more than 10 transitions between protonation states (Figures 3 and S4). To show that phbuilder may also be used to set up more complex systems, we next used it to set up a constant pH simulation for the pentameric ion channel GLIC embedded in a POPC bilayer. We successfully managed to take an embedded structure and use the tool to set up the constant pH input files from there. The subsequent 10 ns simulation showed that the protonation states evolved alongside the atomic coordinates (Figure S5).

### Parameterization of New Titratable Groups

**Figure 4:**
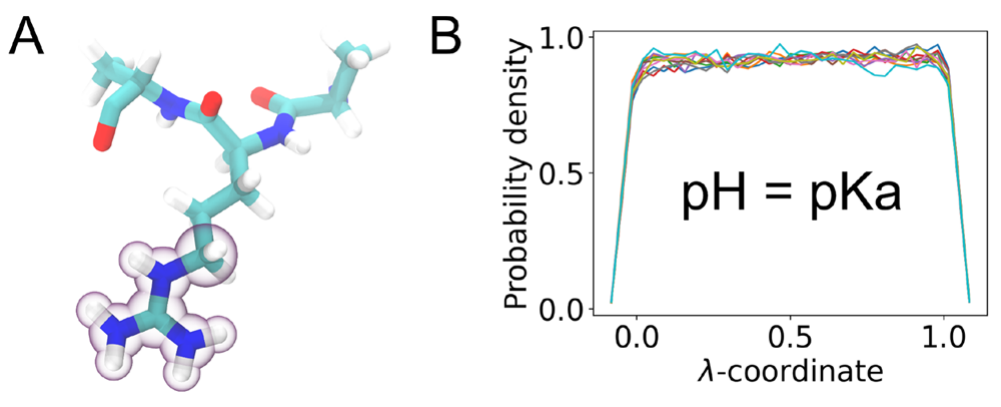
Final parametrization result for arginine. (a) The structure of the tripeptide (titratable atoms highlighted). (B) The distribution of the λ-coordinate for simulations performed at pH = p*K*_a_ and with the biasing barrier set to 0.

Obtaining a sufficiently accurate polynomial fit can sometimes be difficult, which is why we introduced a new two-step approach for the parametrization: (i) aquire an initial polynomial fit by performing a fast, but inaccurate, TI calculation to obtain an approximate correction potential; then, (ii) systematically improve this approximate potential by reweighting the expansion coefficients of the polynomials based on inverse Boltzmann statistics. phbuilder automates the setup of both steps, analyzes the trajectories, and calculates the optimal polynomial coefficients for *V*^MM^. Here, we demonstrate this new parametrization protocol by performing a parametrization for a single-site model of arginine. The fast TI step gave somewhat skewed histograms (Figure S2), which were subsequently corrected by applying a reweighting of the approximate fit using inverse Boltzmann statistics, finally yielding consistently flat λ-distributions (Figures 4 and S2).

## Conclusions

Constant pH MD simulations allow for more accurate modeling of pH dependence compared to traditional MD simulations. While a constant pH MD algorithm was recently implemented in the GROMACS MD package,^27^ it has been proven difficult to efficiently prepare such simulations due to the complex and error-prone nature of their setup process. Here, we have presented phbuilder, a Python-based system builder tool that automates the setup process. It allows the user to set up systems for constant pH MD simulations starting from both initial structures and existing simulation systems. The user can either follow the default protocol, which will generate a generally suitable setup, or provide additional details if some specific tuning is required for the system. phbuilder also allows the user to automatically set up protein titrations or parametrize new titratable groups. We expect that with the help of phbuilder, constant pH MD simulations will be more straightforward to set up and will thereby become more readily accessible to the GROMACS user community.

## Data and Software Availability

Both the phbuilder tool, the GROMACS constant pH MD beta version, and the modified CHARMM36m force field are available for download free of charge at www.gitlab.com/gromacs-constantph. All simulation setup files are available athttps://doi.org/10.5281/zenodo.10468944.

## Supporting Information Available

Example of the clean_after_solvate.py application, the evolution of the λ-coordinates of the test systems, and the reweighting step during parametrization (PDF)
