# Supplementary material for: phbuilder: A Tool for Efficiently Setting up Constant pH Molecular Dynamics Simulations in GROMACS
Source: J Chem Inf Model. 2024 Jan 12;64(3):567–74. doi: 10.1021/acs.jcim.3c01313 (PMC10865341; doi:10.1021/acs.jcim.3c01313)
Supplement: Supplementary file 1 — ci3c01313_si_001.pdf [file ci3c01313_si_001.pdf]

Supplementary Information: phbuilder: a tool for efficiently setting up constant-pH simulations in GROMACS

Anton Jansen[1, †], Noora Aho[2], Gerrit Groenhof[2], Pavel Buslaev[2, †], and Berk Hess[1, †]

[1]Department of Applied Physics and Swedish e-Science Research Center, Science for Life Laboratory, KTH Royal Institute of Technology, 100 44 Stockholm, Sweden
[2]Nanoscience Center and Department of Chemistry, University of Jyväskylä, 40014 Jyväskylä, Finland
[†]Corresponding authors: anton.jansen@scilifelab.se, pavel.i.buslaev@jyu.fi, hess@kth.se

December 5, 2023

Contents

1 Cleaning after solvate **2**

2 Reweighing step of titratable group parameterization **3**

3 Protein and titration dynamics **3**

1 Cleaning after solvate

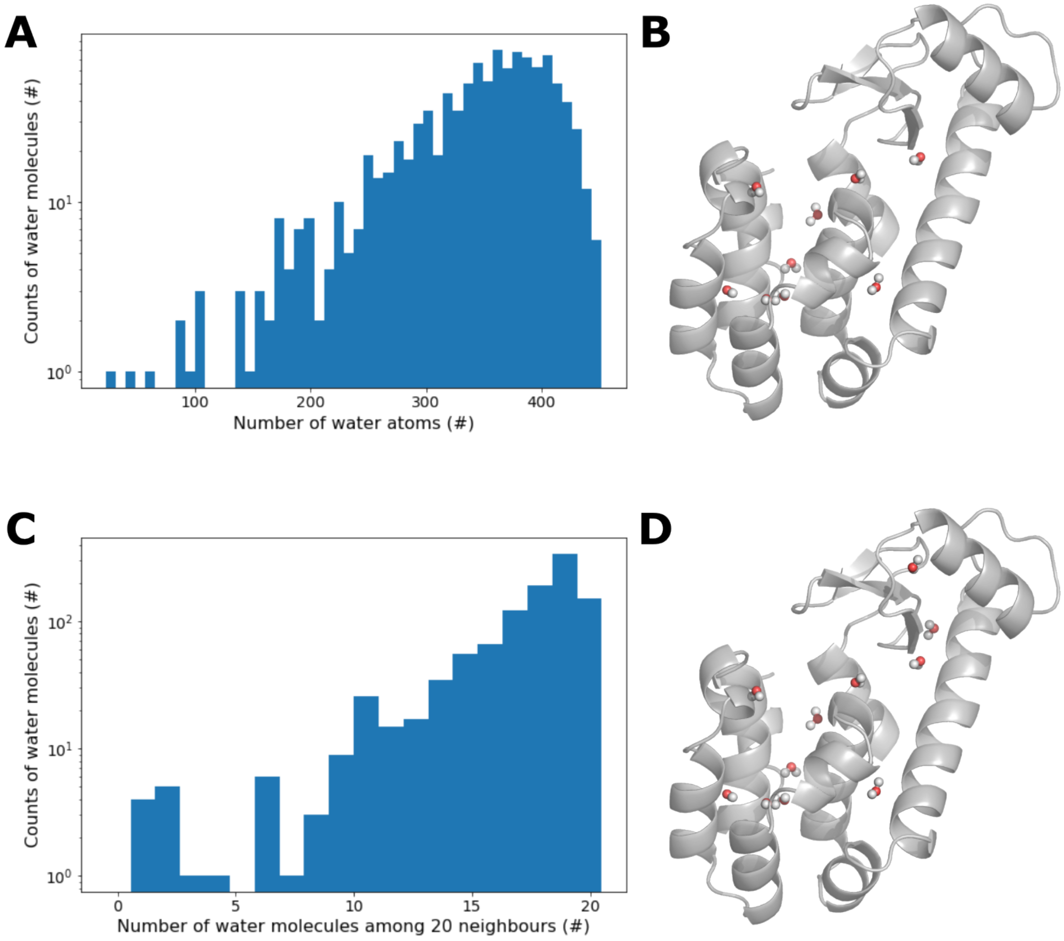

Figure S1: Example of *clean_after_solvate.py* application. Panel A shows the distribution of the number of water atoms within 10 Åsphere of water molecules for lysozyme (PDBID: 2LZM) after *gmx solvate*. There are only a few water molecules for which the number is smaller than 120. Panel B shows the positions of these water molecules. They are placed inside the protein core. Panel C shows the distribution of the number of water molecules among the 20 nearest neighbors for lysozyme (PDBID: 2LZM) after *gmx solvate*. Panel D shows the positions of these water molecules. The majority of them are placed inside the protein core.

2 Reweighing step of titratable group parameterization

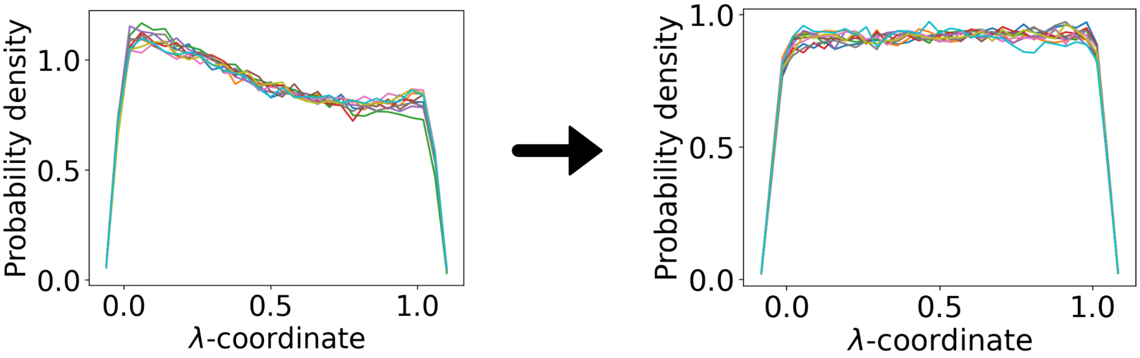

Figure S2: Distribution of λ-coordinates for sampling runs of arginine simulated at pH=pK_{a} and biasing barrier set to 0. The left panel shows distributions obtained from simulations with V^{MM} computed based on a fast thermodynamic integration step. The right panel shows the distributions with V^{MM} obtained after reweighing step.

3 Protein and titration dynamics

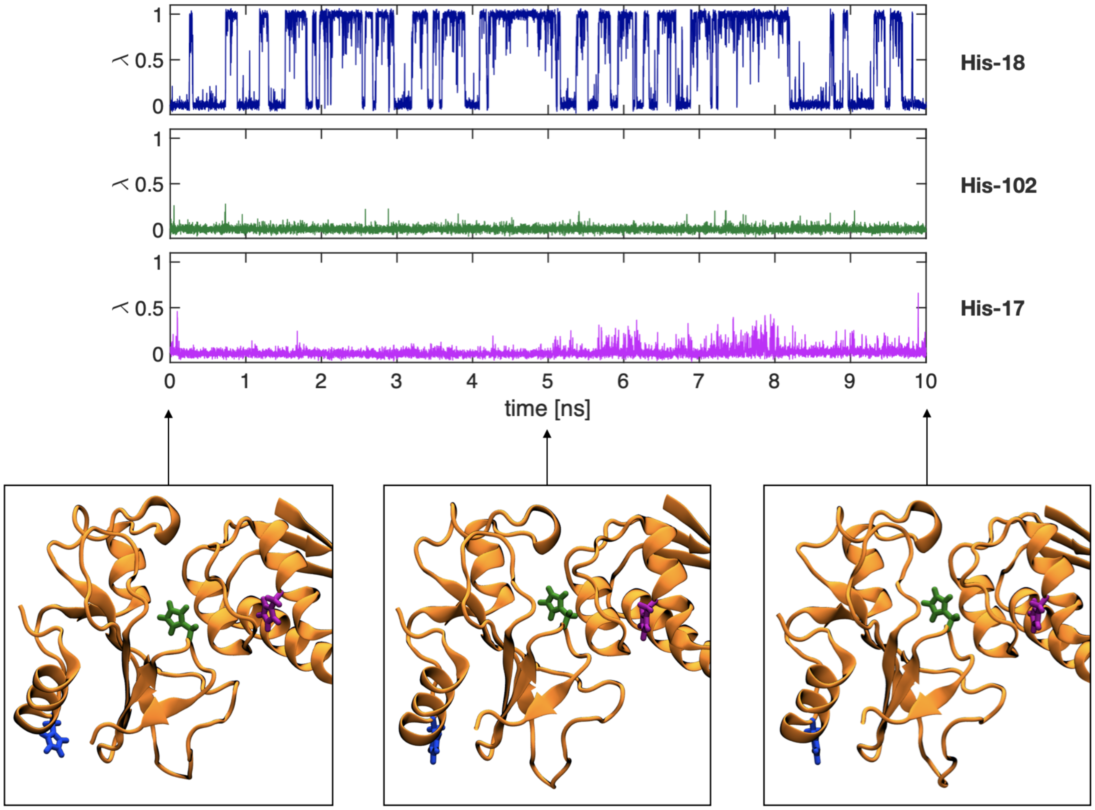

Figure S3: Evolution of the λ-coordinates of histidine residues in time along with snapshots of the trajectory for a 10 ns CpHMD simulation of barnase-barnstar (PDB ID: 1BRS [1]) at pH=7. The upper panel presents the λ-coordinates as a function of simulation time for His-18 and His-102 of barnase and His-17 of barstar, and the lower panel the snapshots at 0, 5, and 10 ns. In the snapshots, the titratable groups are shown with the stick representation, and the colors of the groups match with the λ-coordinate plots.

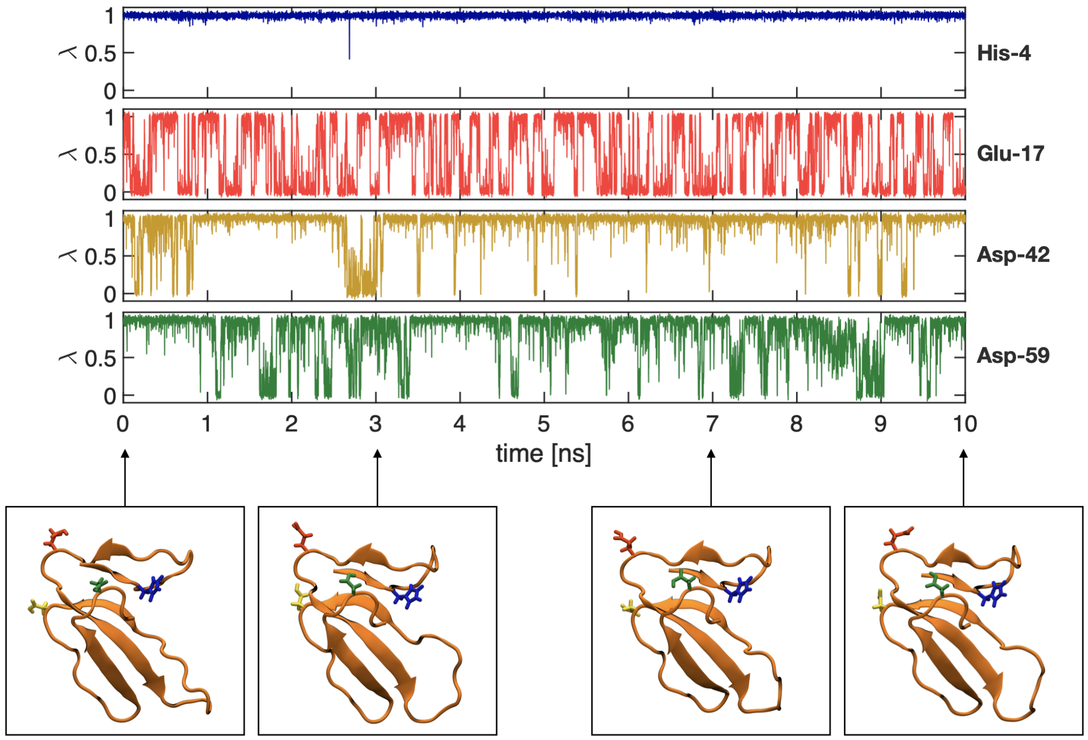

Figure S4: Evolution of the λ-coordinates in time along with snapshots of the trajectory for a 10 ns CpHMD simulation of cardiotoxin V (PDB ID: 1CVO [2]) system setup starting from the already prepared system, at pH=4. The upper panel presents the λ-coordinates as a function of simulation time for His-4, Glu-17, Asp-42, and Asp-59, and the lower panel the snapshots at 0, 3, 7, and 10 ns. In the snapshots, the titratable groups are shown with the stick representation, and the colors of the groups match with the λ-coordinate plots.

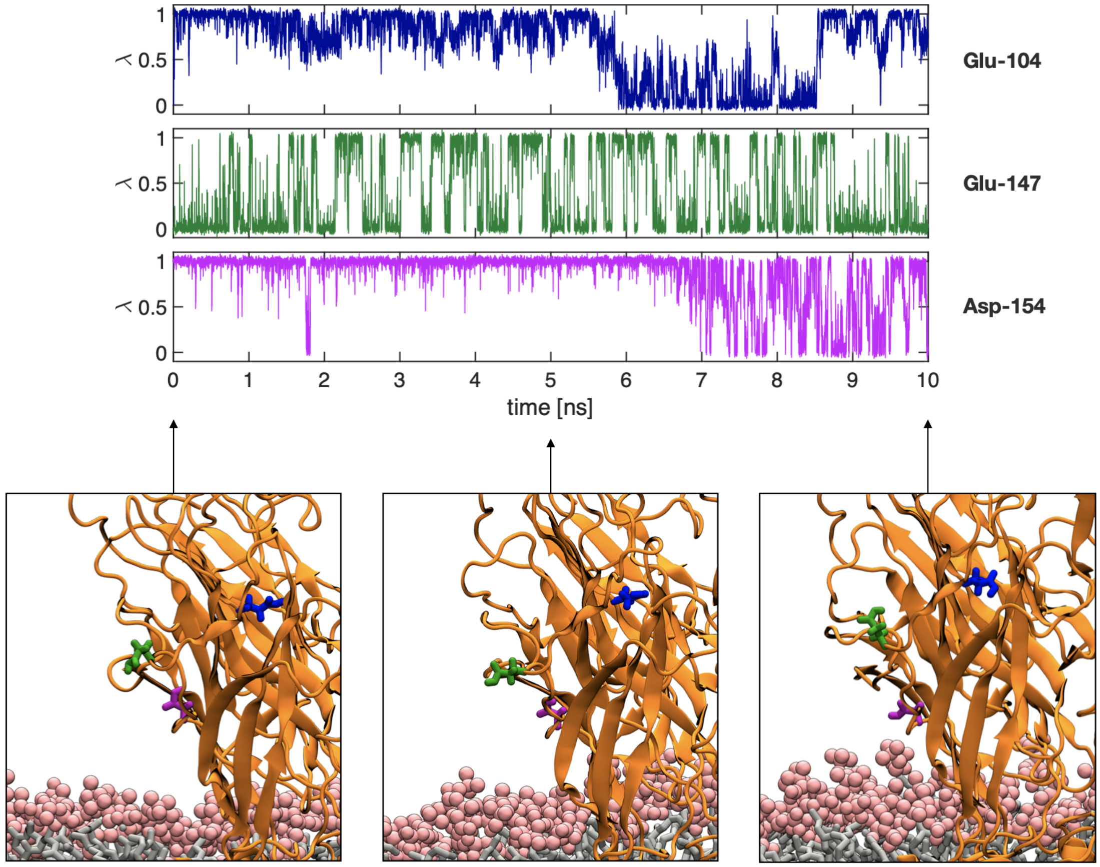

Figure S5: Evolution of few λ-coordinates of selected residues in time along with snapshots of the trajectory for a 10 ns CpHMD simulation of GLIC ion channel (PDB ID: 6ZGD [3]) at pH=7. The upper panel presents the λ-coordinates as a function of simulation time for Glu-104, Glu-147, and Asp-154 of subunit A, and the lower panel the snapshots at 0, 5, and 10 ns. In the snapshots, the titratable groups are shown with the stick representation, and the colors of the groups match with the λ-coordinate plots. The membrane is represented as pink spheres (lipid headgroup atoms) and gray sticks (lipid tails).

References

[1] Ashley M Buckle, Gideon Schreiber, and Alan R Fersht. Protein-protein recognition: Crystal structural analysis of a barnase-barstar complex at 2.0-. ang. resolution. *Biochemistry*, 33(30):8878–8889, 1994.

[2] Arun K Singhal, Kun Yi Chien, Wen Guey Wu, and Gordon S Rule. Solution structure of cardiotoxin v from naja naja atra. *Biochemistry*, 32(31):8036–8044, 1993.

[3] Urška Rovšnik, Yuxuan Zhuang, Björn O Forsberg, Marta Carroni, Linnea Yvonnesdotter, Rebecca J Howard, and Erik Lindahl. Dynamic closed states of a ligand-gated ion channel captured by cryo-em and simulations. *Life Science Alliance*, 4(8), 2021.
